# Ostriches Sleep like Platypuses

**DOI:** 10.1371/journal.pone.0023203

**Published:** 2011-08-24

**Authors:** John A. Lesku, Leith C. R. Meyer, Andrea Fuller, Shane K. Maloney, Giacomo Dell'Omo, Alexei L. Vyssotski, Niels C. Rattenborg

**Affiliations:** 1 Max Planck Institute for Ornithology, Seewiesen, Germany; 2 University of the Witwatersrand, Johannesburg, South Africa; 3 University of Western Australia, Crawley, Australia; 4 Ornis Italica, Rome, Italy; 5 University of Zurich/ETH Zürich, Zurich, Switzerland; McGill University, Canada

## Abstract

Mammals and birds engage in two distinct states of sleep, slow wave sleep (SWS) and rapid eye movement (REM) sleep. SWS is characterized by slow, high amplitude brain waves, while REM sleep is characterized by fast, low amplitude waves, known as activation, occurring with rapid eye movements and reduced muscle tone. However, monotremes (platypuses and echidnas), the most basal (or ‘ancient’) group of living mammals, show only a single sleep state that combines elements of SWS and REM sleep, suggesting that these states became temporally segregated in the common ancestor to marsupial and eutherian mammals. Whether sleep in basal birds resembles that of monotremes or other mammals and birds is unknown. Here, we provide the first description of brain activity during sleep in ostriches (*Struthio camelus*), a member of the most basal group of living birds. We found that the brain activity of sleeping ostriches is unique. Episodes of REM sleep were delineated by rapid eye movements, reduced muscle tone, and head movements, similar to those observed in other birds and mammals engaged in REM sleep; however, during REM sleep in ostriches, forebrain activity would flip between REM sleep-like activation and SWS-like slow waves, the latter reminiscent of sleep in the platypus. Moreover, the amount of REM sleep in ostriches is greater than in any other bird, just as in platypuses, which have more REM sleep than other mammals. These findings reveal a recurring sequence of steps in the evolution of sleep in which SWS and REM sleep arose from a single heterogeneous state that became temporally segregated into two distinct states. This common trajectory suggests that forebrain activation during REM sleep is an evolutionarily new feature, presumably involved in performing new sleep functions not found in more basal animals.

## Introduction

Mammals engage in two types of sleep, slow wave sleep (SWS) and rapid eye movement (REM) sleep. SWS is characterized by slow, high amplitude brain waves [1], while REM sleep is characterized by fast, low amplitude waves (reflecting brain activation), rapid eye movements, and reduced muscle tone [2]. Unlike SWS, which is initiated and maintained by the forebrain, REM sleep-related cortical activation, rapid eye movements, and reduced muscle tone are generated by the brainstem [2], [3]. Interestingly, the cortex of monotremes (platypuses and echidnas), the most basal (or ‘ancient’) group of living mammals, shows only SWS-like slow waves during sleep [4]–[6], [ but see 7]. Furthermore, during sleep in the platypus (*Ornithorhynchus anatinus*), cortical slow waves occur with REM sleep-like rapid eye movements and reduced muscle tone [8]. This suggests that REM sleep at the level of the brainstem and SWS in the cortex were present in the most recent common ancestor to all mammals, and that REM sleep with cortical activation evolved only after the appearance of the marsupial/eutherian lineage [5], [9]. Alternatively, the unusual brain activity of sleeping monotremes may reflect an evolutionary loss of REM sleep with cortical activation [10].

One way to distinguish between these possibilities would be to characterize REM sleep in reptiles, the sister-group to mammals. However, reptiles do not exhibit the neuronal activity observed in the brainstem during REM sleep in mammals [11], including monotremes [12], nor do they show cortical signs of REM sleep and SWS [11], [13], [14]. Alternatively, animals that independently evolved SWS and REM sleep may provide insight into the evolution of REM sleep by revealing recurring evolutionary patterns. Because birds are the only animals outside of mammals to engage in SWS and REM sleep, only birds can provide such insight. However, whether basal birds exhibit brain activity during sleep that resembles that of monotremes or other mammals and birds is unknown [15]–[19]. Here, we provide the first description of sleep electrophysiology in ostriches (*Struthio camelus*), a member of the most basal group of living birds. We found that the brain activity of ostriches during sleep is unique, and most closely resembles that of the distantly-related monotremes, revealing a recurring sequence of steps in the evolution of REM sleep.

## Methods

Six female adult ostriches (82±4 kg, mean ± s.e.m.) were purchased from a farm in Free State, South Africa, and transported to the Lichtenburg Game Breeding Center, South Africa (26°06′S, 26°10'E) for study. The study was conducted in February and March 2009 (southern hemisphere summer). The birds were implanted with electrodes for measuring brain waves of the hyperpallium (electroencephalogram, EEG), eye movements (electrooculogram, EOG), neck muscle tone (electromyogram, EMG) and a thermistor for brain temperature using standard aseptic techniques by experienced surgeons (see Text S1 for details). EEG, EOG and EMG electrodes terminated at a plug housed in a head-mounted aluminum box (length×width×height: 44×24×32 mm). The plug connected to an upgraded version of a logger (Neurologger) previously used for recording the EEG of birds [20] (Text S1). A 3-dimensional accelerometer on the Neurologger recorded acceleration as a positive or negative deflection depending on the direction of the movement along each of the three axes; the magnitude of the deflection was proportional to the acceleration. Temperature was recorded via a thermistor in the brain connected to a logger positioned subcutaneously in the neck [21] (Text S1). All methods were approved by the National Zoological Gardens of South Africa (P08/22) and the Animal Ethics Screening Committee at the University of the Witwatersrand (2008/45/05), and adhere to the NIH standards regarding the care and use of animals in research.

**Figure 1:**
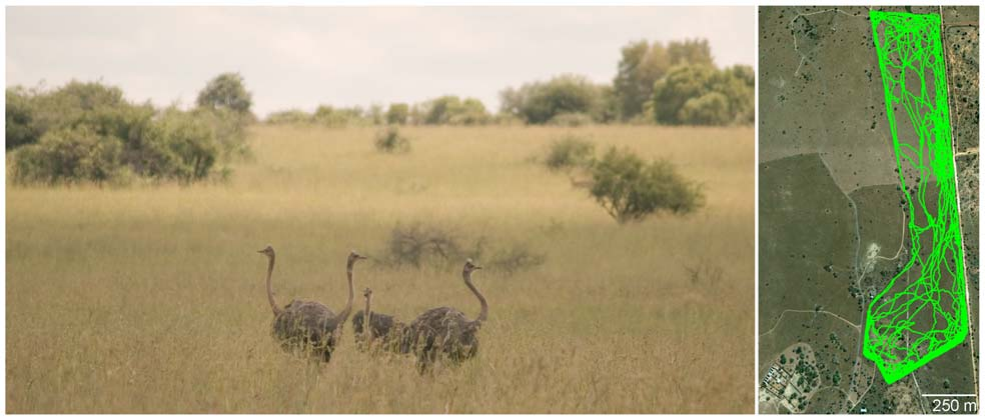
Four of the ostriches in the naturalistic reserve in South Africa (*left*). Photograph by J.A.L. Movement data (green tracks, sampled once per second) from one ostrich for its first 8 d in the reserve (*right*); outline of the tracks shows the boundary of the reserve. Satellite map from Google Earth (www.google.com/earth).

The recordings were conducted at two locations. First, the ostriches were group-housed in an outdoor enclosure (5×5 m) with occasional access to a connecting enclosure of similar size. Grass (*Eragrostis* spp.), alfalfa (*Medicago sativa*), pelleted ostrich food and clean water were available *ad libitum*. The main enclosure was monitored using 8 video cameras equally spaced along the perimeter, and an infrared illuminator in each corner provided light (850 nm) for nighttime recordings. These video recordings were used to establish relationships between specific behaviors and the electrophysiological and accelerometer signals. After 7–10 d, the ostriches were moved to a large (51 ha) naturalistic reserve less than 1 km away (Figure 1). The reserve had a floral assemblage characteristic of South African savannah (or Highveld) and large herbivores that are sympatric with ostriches in the wild (e.g., blesbok, *Damaliscus pygargus phillipsi*; impala, *Aepyceros melampus*; roan antelope, *Hippotragus equines*). Ostriches occupied the full area of the reserve, as determined by a GPS logger attached to the leg of each bird for their first 10 days in the camp (Figure 1). These naturalistic recordings continue the recent push for EEG-based sleep research to move into more wild environments [22], as some aspects of normal physiology may not be reflected in the laboratory [19], [22]–[26].

EEG, EOG, EMG and head movements were recorded from all ostriches for between 0.7 to 18.6 d total (9.2±2.8 d, mean ± s.e.m.). Signals were downsampled from 800 Hz to 200 Hz for visualization and analysis in Somnologica Science v. 3.3.1 (Embla®, www.embla.com). One undisturbed 24 h day in the reserve (∼13L∶11D) was visually scored for wakefulness, SWS and REM sleep using 4 s epochs. Epochs that contained more than one state were scored according to the state occupying the majority of that epoch. This undisturbed day was characterized by unexceptional temperatures (black globe temperature, day: 29.7±0.1°C, night: 14.6±0.7°C), little-to-no wind (wind speed, day: 0.80±0.29 m/s, night: 0.05±0.03 m/s), and no rain, as measured by a weather station adjacent to the reserve. Brain temperature was recorded successfully from 5 of the 6 ostriches throughout the entire study. To investigate the relationship between brain state and temperature, we compared brain temperature at night during wakefulness to that during sleep. Because the logger recorded brain temperature instantaneously at the top of every second minute, only bouts of wakefulness and sleep that occupied the entire 2 min period immediately before temperature was recorded were included in this analysis. Brain temperature during REM sleep could not be calculated reliably as episodes of REM sleep rarely met this criterion. Data were analyzed with one-way repeated measures analysis of variance (rmANOVA) or paired t-tests using SYSTAT 10 (©SPSS, Inc., www.systat.com).

## Results

**Figure 2:**
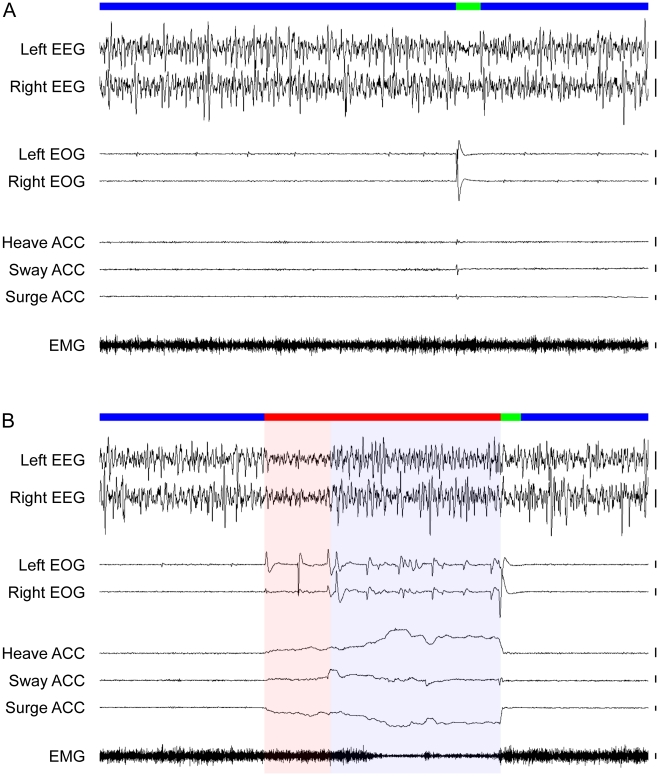
(A) Representative slow wave sleep (SWS, blue bar) in the ostrich characterized by high amplitude, slow waves in the electroencephalogram (EEG), the absence of rapid eye movements (measured via electrooculogram, EOG) and head movements (accelerometer, ACC), and moderate muscle tone (electromyogram, EMG). Here, SWS is interrupted by a brief awakening (green bar) characterized by low amplitude, high frequency EEG activity, and a fast (200 ms) lateral sweep of the head, perhaps as a quick scan of the local environment, followed by a re-entrance into SWS. (B) Representative rapid eye movement (REM) sleep (red bar). Note that the EEG during REM sleep shows either activation (red shading) or slow waves (blue shading). Irrespective of the type of EEG activity, rapid eye movements, a forward falling and swaying head with moderate-to-low muscle tone occurred invariably during REM sleep in the ostrich. Heave ACC: movement along the dorso-ventral axis with a positive slope denoting downward movement, Sway ACC: lateral axis with positive denoting movement to the right, Surge ACC: anterior-posterior axis with negative denoting movement forward. Vertical bars to the right of each EEG, EOG and EMG trace denote 100 µV, and 100 milli g-forces to the right of each ACC trace. Trace duration: 60 s.

An awake ostrich had both eyes open and was generally walking, feeding or preening. Not surprisingly, during such periods, neck muscle tone was highest and eye movements were common. Sleep followed with the cessation of these waking activities. During SWS, ostriches typically sat motionlessly with their necks held periscopically above the ground; both eyes were always open though without movement (Movie S1). Consequently, an ostrich in SWS did not look like a typical sleeping animal and instead gave the impression of an alert bird. This wake-like sleep posture may explain why sleep is rarely reported in studies on time budgets and activity patterns in wild ostriches [27], [28]. SWS with open eyes has been reported in other avian [29]–[32] and mammalian [33], [34] species, and may allow for visual processing concurrent with sleep [32], [35]. During SWS, the EEG showed slow waves (Figure 2A, Figure S1) like those recorded from other birds engaged in SWS [36]–[38]. The amplitude of slow waves during SWS were largely symmetric between the hemispheres, although short asymmetries were observed periodically. The magnitude of these asymmetries resembled that observed in some other birds [31], [32].

**Figure 3:**
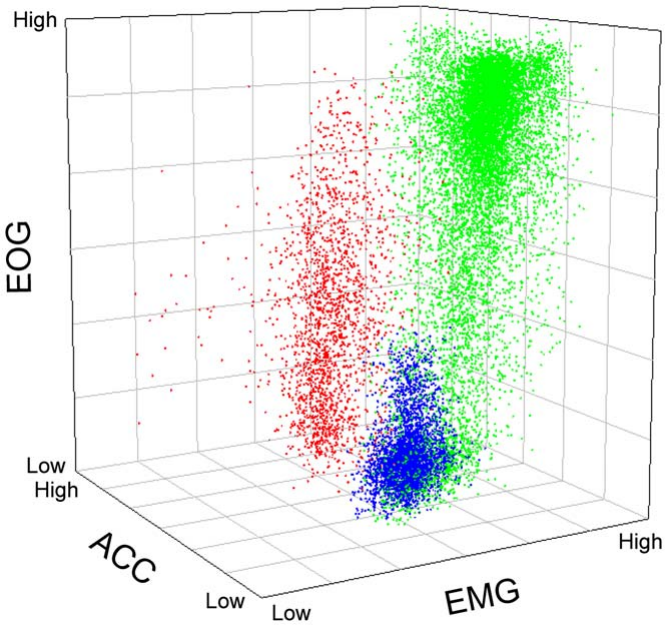
Plot of data from an ostrich illustrating the distinctiveness of wakefulness (green), slow wave sleep (SWS, blue) and rapid eye movement (REM) sleep (red) based on differences in eye movements (measured via electrooculogram, EOG), head movements (accelerometer, ACC) and neck muscle tone (electromyogram, EMG). SWS is associated with few eye movements, a relatively motionless head and moderate muscle tone; REM sleep is associated with rapid eye movements, head movements and moderate-to-low muscle tone. During wakefulness, muscle tone is generally highest with large head and eye movements. Variables calculated as the logarithm of power density (EOG: 0.4–9.8 Hz using the larger value between the left and right eye for each epoch, surge axis of the ACC: 0.0–9.8 Hz, EMG: 9.8–69.9 Hz). See Figure S2 for the three 2-dimensional plots that constitute this 3-dimentional figure.

The transition from SWS to REM sleep was marked by bilateral eye closure, rapid eye movements, and a forward falling head (Movie S1, Figure 2B, Figure S1). As in owls [29], [30] and some ruminating mammals [33], bilateral eye closure was observed only in conjunction with REM sleep. In ostriches, the drooping and swaying head movements that accompanied REM sleep were readily distinguishable from movements occurring during wakefulness (Figure 3, Figure S2). In extreme cases, the head fell to the ground [see also 39], [40]. This behavioral correlate of REM sleep has been observed in wild ostriches, where it was attributed to drowsiness:

“Closing its eyes, a tired Ostrich would slowly tilt its head downward and, after a while, jerk it up just to droop it again.” [41]

Interpreting this behavior as belonging to a drowsy animal is understandable given the alert-like sleep posture of an ostrich engaged in SWS. These REM sleep-related head movements have also been described in a close relative of the ostrich, the greater rhea (*Rhea americana*) [42]. Concomitant with this REM sleep behavior in ostriches, muscle tone was generally lower than during SWS (Figure 2B, Figures S1,S2). The end of an episode of REM sleep was almost always marked by a rapid rise of the head, cessation of rapid eye movements, and restoration of wake-like or SWS-like muscle tone (depending on the state entered next) (Figure 2B, Figure S1). Thus, the EOG and accelerometer signals served as well-defined ‘bookends’ to an episode of REM sleep. Within these ‘bookends’, the EEG showed SWS-like slow waves that alternated with REM sleep-like activation (Figure 2B, Figure S1). This mixed REM sleep state was identified in all ostriches. REM sleep with activation and REM sleep with slow waves could both occur with rapid eye movements, reduced muscle tone, and head movements; indeed, REM sleep with slow waves could occur with rapid eye movements and the lowest muscle tone (Figure 2B, Figure S1). This and the fact that the amplitude of slow waves during an episode of REM sleep was generally stable (e.g., Figure 2B, Figure S1), suggest that these slow waves do not simply reflect transitions into and out of REM sleep; in all other birds studied, such transitions are rapid (<2 s) [31], [36], [37], [43]–[50]. Indeed, such an unusual REM sleep state has not been reported previously in any bird, despite many studies of avian sleep, on phylogenetically diverse species, that employed comparable EEG, EOG and EMG recording techniques [31], [36], [37], [43]–[50].

**Figure 4:**
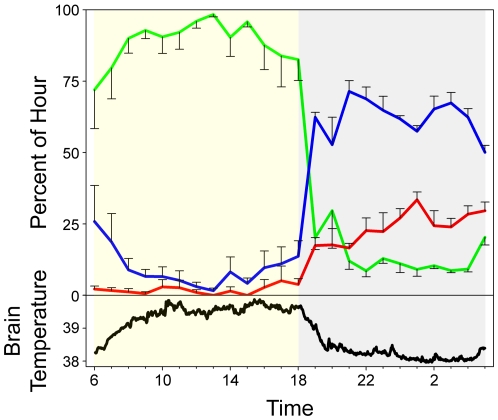
The percentage of time (mean, s.e.m.) spent in wakefulness (green), slow wave sleep (SWS, blue) and rapid eye movement (REM) sleep (red) for each hour of the day (sunrise-to-sunset, yellow shading) and night (grey shading). Brain temperature (°C) is given at the bottom of the panel.

Based on the electrophysiological and accelerometer signals recorded from the animals in the reserve, ostriches spend 88.6±1.7% (mean ± s.e.m.) of the day and 13.8±1.8% of the night awake (Figure 4). This daytime value is similar to the amount of unequivocal wakefulness (i.e., activity) reported for ostriches in the wild [51], [52]. Such diurnality was reflected in brain temperature, which was significantly higher during the day (39.4±0.1°C) than during the night (38.3±0.1°C, P<0.001) (Figure 4). Ostriches spend 9.5±1.5% of the day and 62.2±2.1% of the night in SWS (Figure 4). The amount of SWS decreased across the night (rmANOVA on ‘time of night’: F = 2.791, df = 10,30, P = 0.014) (Figure 4), a pattern that has been observed in other birds [31], [38], [49]. The brain was significantly cooler when in SWS after sunset (38.2±0.1°C) than when awake after sunset (39.2±0.3°C, P = 0.047); however, circadian effects on brain temperature cannot be discounted, as long (≥2 min) bouts of SWS and wakefulness were rare before and after astronomical twilight, respectively. REM sleep occupied 1.9±0.9% of the day and 24.0±0.9% of the night (or 26.3±1.3% of 24 h total sleep time) (Figure 4), the most reported for any bird [18], [19], [53]. Although the amount of REM sleep increases across the night in other birds [31], [38], [49], [50], the mean increase in ostriches (Figure 4) did not reach statistical significance (rmANOVA on ‘time of night’: F = 1.757, df = 10,30, P = 0.113) nor did the mean increase in REM sleep/total sleep time (F = 1.974, df = 10,30, P = 0.073), perhaps due to the small sample size. Episodes of REM sleep, typically less than 10 s in duration in other birds [36], [37], [50], [54], lasted 27±7 s on average in ostriches, and could last up to 5 min (2.3±0.9 min, mean maximum ± s.e.m.), the longest reported for any bird.

## Discussion

Ostriches exhibit a heterogeneous REM sleep state characterized by eye closure, rapid eye movements, reduced muscle tone, and a forward falling head, occurring with forebrain activity that flips between REM sleep-like activation and SWS-like slow waves. To our knowledge, such a state has not been reported previously in any bird. Ostriches also have the longest REM sleep episodes, and more REM sleep overall, than any other avian species. The unusual REM sleep state of ostriches is unlikely to be related to their large size *per se*, because the Emperor penguin (*Aptenodytes forsteri*), the next largest species studied (∼28 kg), shows REM sleep typical of other birds [37]. Moreover, REM sleep occupied 13% of sleep time, and the duration of REM sleep episodes was less than 10 s in penguins [37], values typical of small birds [18], [19], [36], [38], [50], [53], [54].

How might the ostrich brain initiate this heterogeneous REM sleep state? In mammals, REM sleep-related forebrain activation, rapid eye movements, and reduced muscle tone are generated by the brainstem [2]. In mammals [2], [3] and birds [55], forebrain activation arises via the excitatory action of ascending cholinergic REM sleep-on neurons in the rostral pons of the brainstem. Flipping between activation and slow waves during REM sleep in ostriches might reflect variation in the strength of signals from ascending REM sleep-on neurons that promote activation [56] and SWS-generating mechanisms of the ventrolateral preoptic nucleus [57], [58] or those intrinsic to the forebrain [59]–[61]. If true, then these competing effects appear to occur independently from variation in the strength of descending REM sleep-on neurons that reduce muscle tone [56], because the lowest tone could occur either when the hyperpallium was activated or showed slow waves. An investigation combining EEG and recordings of neuronal activity in the brainstem and ventrolateral preoptic nucleus might reveal the source of the unique REM sleep state in ostriches.

The slow wave component of the REM sleep state described here in ostriches resembles that observed in monotremes. Indeed, monotremes are the only other animals known to engage in slow waves during a state which would otherwise be unequivocally identified as REM sleep [4]–[6], [8], [9], [12]. Concurrent with slow waves in the cortex, platypuses exhibit REM sleep-like rapid eye movements, reduced muscle tone, and twitches of the head and bill [8]. If one calculates the amount of REM sleep as periods with rapid eye movements and reduced muscle tone, then platypuses have more REM sleep than any other mammal [8], [9], [62], [63], just as ostriches have more REM sleep than any other bird using similar criteria.

Why might ostriches sleep like platypuses? There appear to be few traits unique to ostriches and monotremes that could explain such an unusual REM sleep state. However, the fact that monotremes and ostriches are both members of the most basal group within their respective lineage [64], [65], suggests that this type of REM sleep may reflect an early stage in the evolution of REM sleep. Although other (yet unknown) factors may explain the similarities between ostrich and monotreme REM sleep, it is remarkable that of all the species studied (c. 100 mammals [9], [63] and 30 birds [15]–[19]) *only* species of the most basal lineages exhibit such a state. The absence of REM sleep in the brainstem and cortex of turtles [11], suggests that the aspects of REM sleep common to monotremes and ostriches arose independently in the most recent common ancestor to all mammals and again in ancestral birds (although an analogous study on a crocodilian, as the closest living relative to modern birds, would help clarify the evolutionary origin of the REM sleep state described here in ostriches). In mammals, forebrain activation during REM sleep (or ‘classical’ REM sleep) evolved in the common ancestor of marsupial and eutherian mammals, as monotremes may not engage in a comparable state. In birds, ‘classical’ REM sleep was apparently present, at least to some extent, in the ancestor to all living birds, but alternates with the more basal, monotreme-like REM sleep state. It is possible that earlier birds may have slept exclusively like monotremes. This evolutionary scenario suggests a recurring sequence of steps in the evolution of REM sleep shared by mammals and birds in which SWS and REM sleep arose as a single heterogeneous state that became temporally segregated into distinct SWS and REM sleep with forebrain activation. Furthermore, it suggests that, as an evolutionarily new feature of sleep, forebrain activation during ‘classical’ REM sleep may support shared sleep functions not found in more basal animals. Identifying the functional significance of this evolutionary pattern is an important avenue for future research.

## Supporting Information

Figure S1 **(A–H) Electroencephalogram (EEG) of the left and right hyperpallia, electrooculogram (EOG) of the left and right eye, the three axes (heave, sway and surge) of the head-mounted accelerometer (ACC), and electromyogram (EMG) of the nuchal muscle showing slow wave sleep (SWS, blue bar), rapid eye movement (REM) sleep (red bar) and wakefulness (green bar) in the ostrich.** See main text for a description of each state. These figures illustrate the well-defined nature of an episode of REM sleep, as well as demonstrate the variation in EEG and EMG activity during REM sleep. Heave ACC: movement along the dorso-ventral axis with a positive slope denoting downward movement, Sway ACC: lateral axis with positive denoting movement to the right, Surge ACC: anterior-posterior axis with negative denoting movement forward. Vertical bars to the right of each EEG, EOG and EMG trace denote 100 µV, and 100 milli g-forces to the right of each ACC trace. Trace duration: 60 s.
(PDF)

Figure S2 **The three 2-dimensional plots that constitute the 3-dimensional** **Figure 3** **in the main article.** (reprinted here in the bottom left corner). These plots illustrate the distinctiveness of wakefulness (green), slow wave sleep (SWS, blue) and rapid eye movement (REM) sleep (red) based on differences in eye movements (measured via electrooculogram, EOG), head movements (accelerometer, ACC) and neck muscle tone (electromyogram, EMG). Variables calculated as the logarithm of power density (EOG: 0.4–9.8 Hz using the larger value between the left and right eye for each epoch, surge axis of the ACC: 0.0–9.8 Hz, EMG: 9.8–69.9 Hz).
(TIF)

Movie S1 **Video showing the behavioral correlates of slow wave sleep (SWS) and rapid eye movement (REM) sleep in the ostrich.** SWS is characterized by open eyes and a vertically-held head; REM sleep is characterized by bilateral eye closure and a drooping head.
(MP4)

Text S1 **Supporting Material.**
(PDF)
