# Supplementary figures and images for: Ostriches Sleep like Platypuses
Source: PLoS One. 2011 Aug 24;6(8):e23203. doi: 10.1371/journal.pone.0023203 (PMC3160860; doi:10.1371/journal.pone.0023203)

A

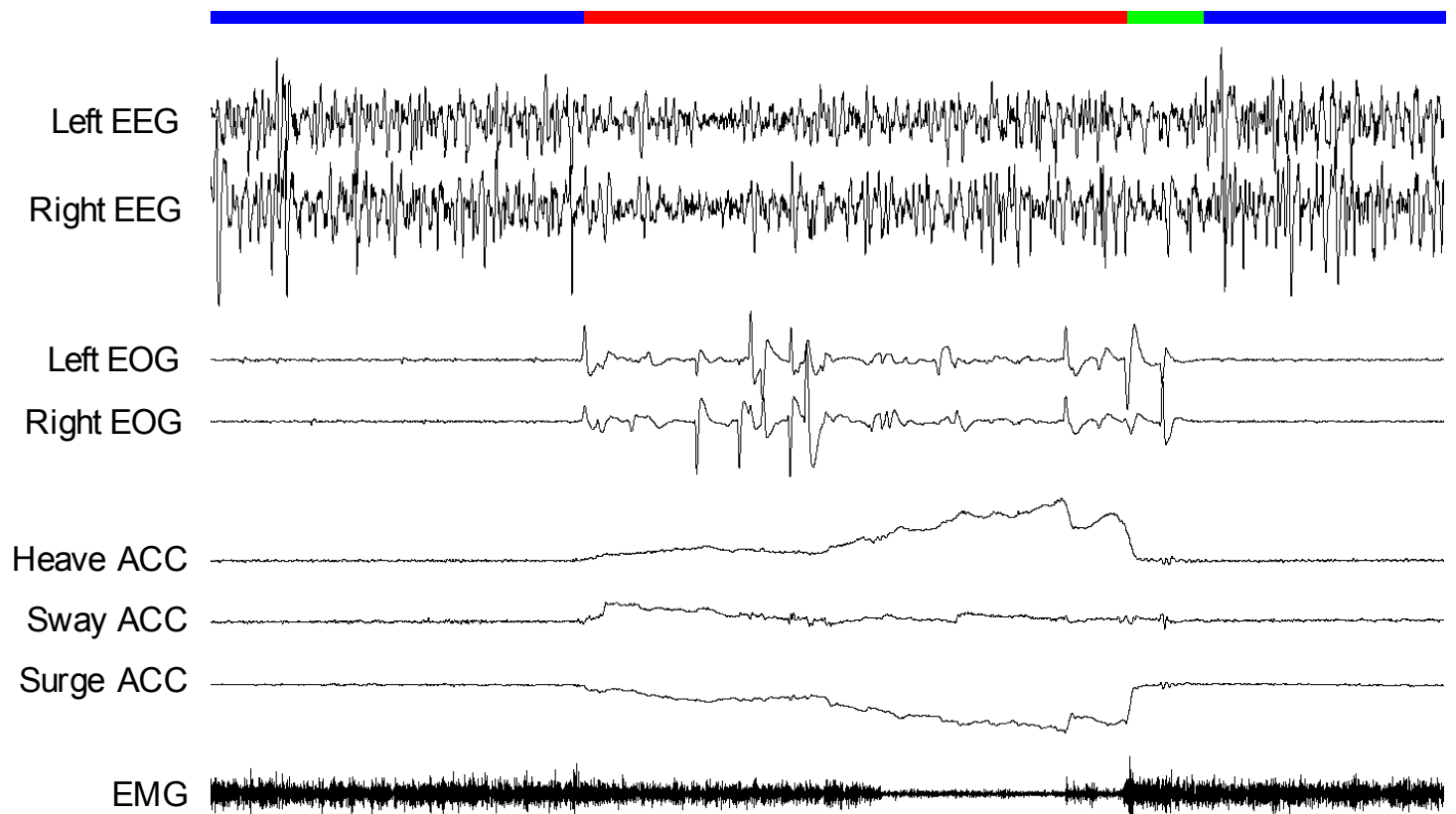

B

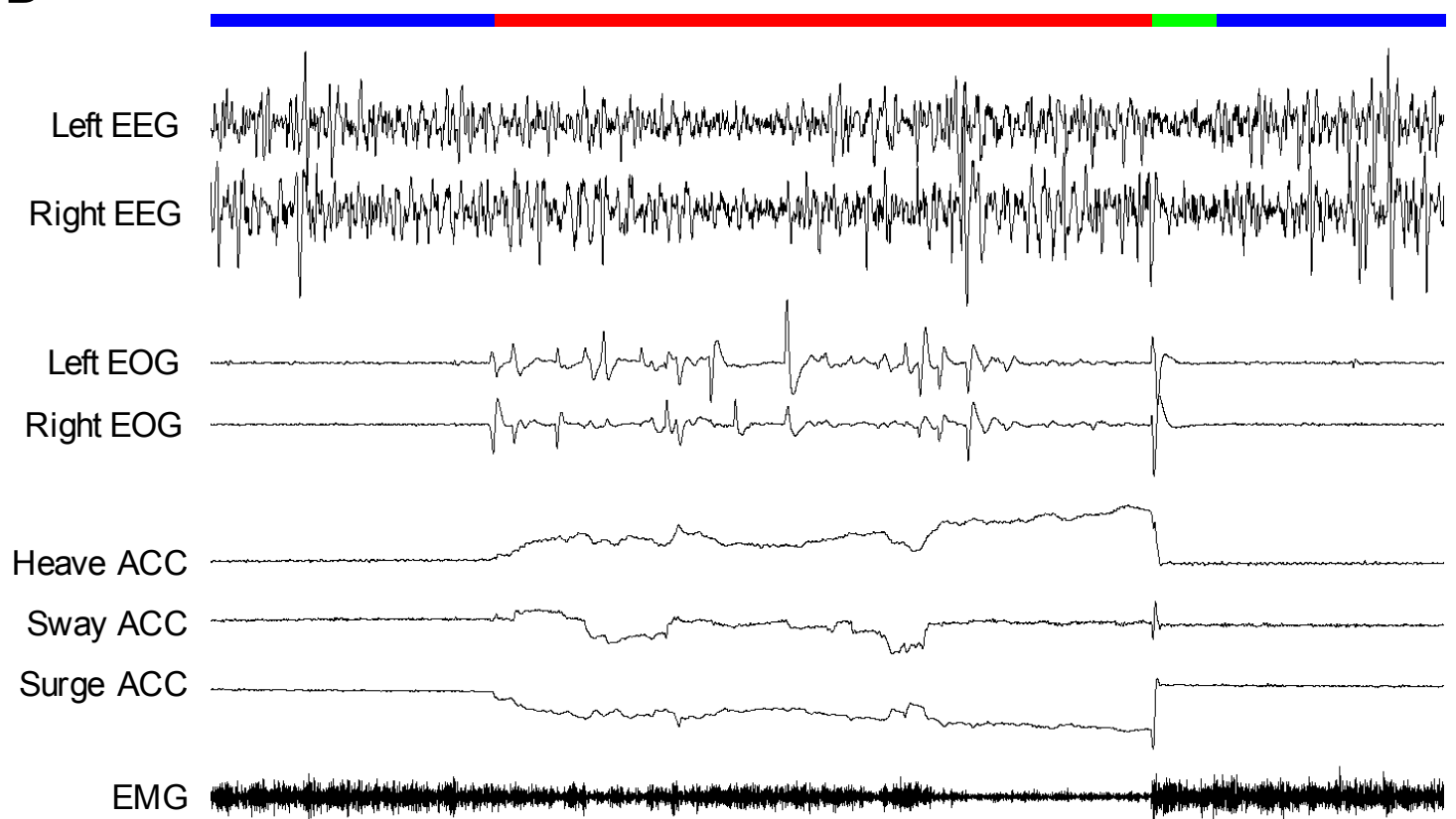

C

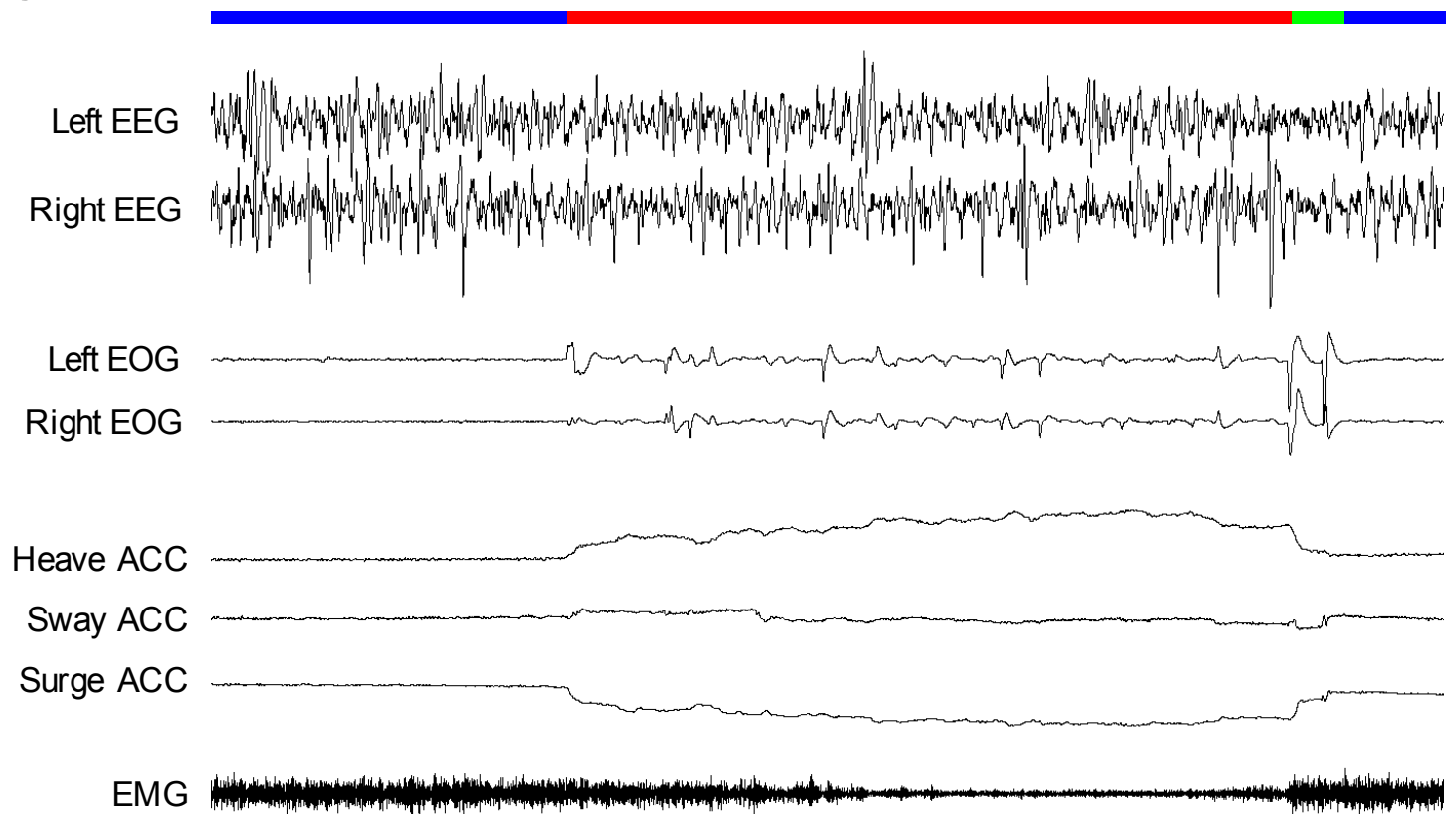

D

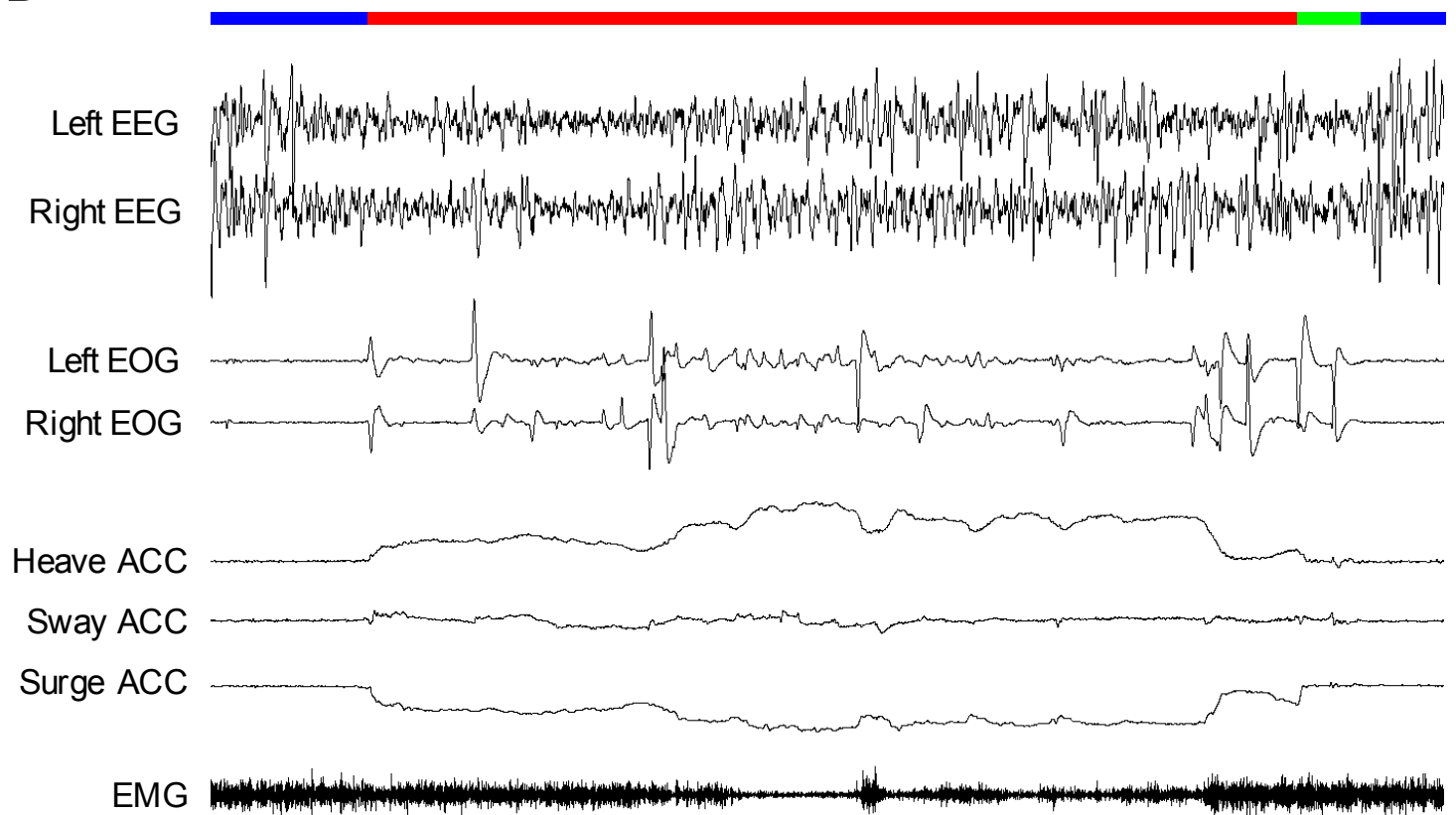

E

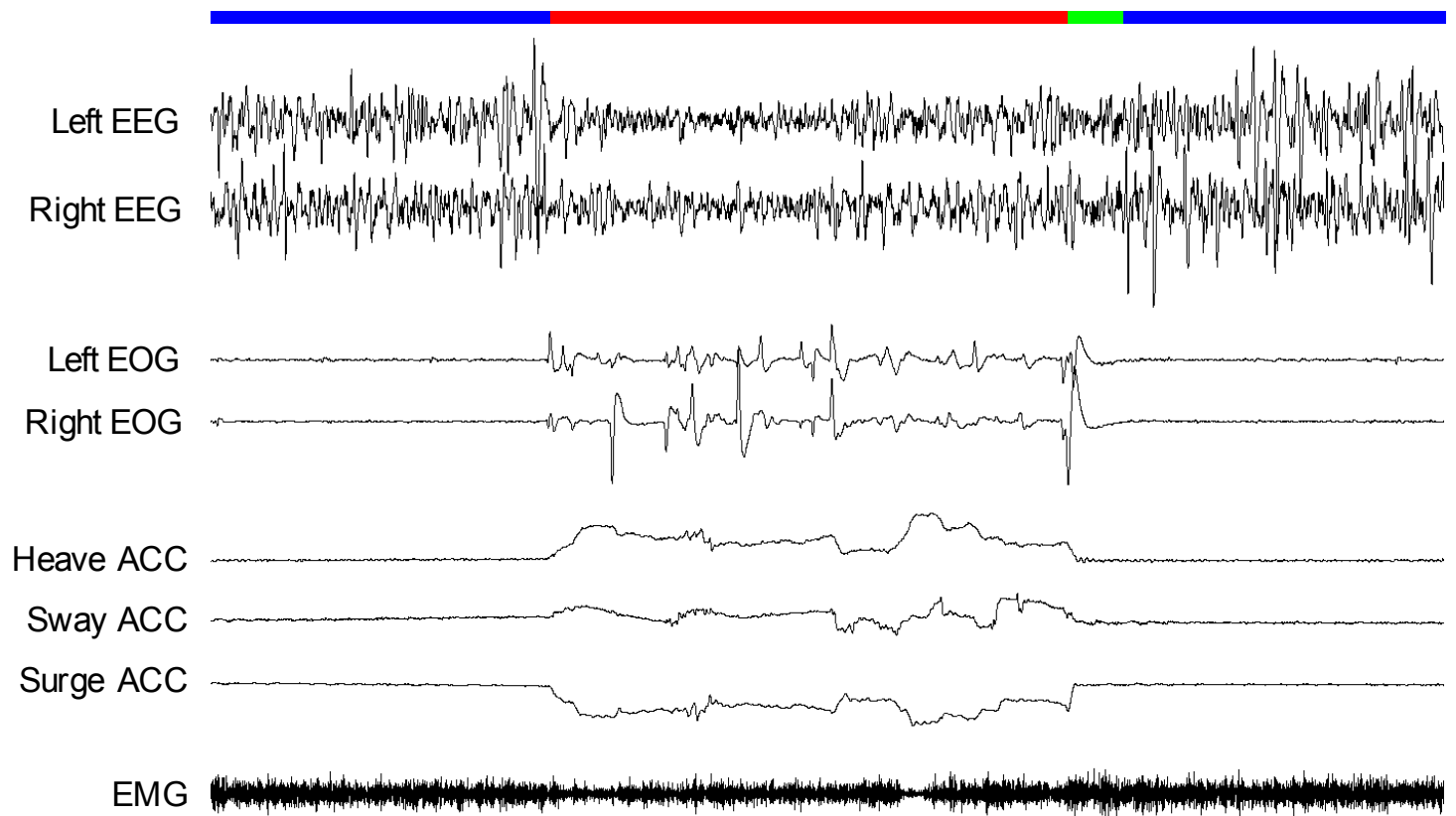

F

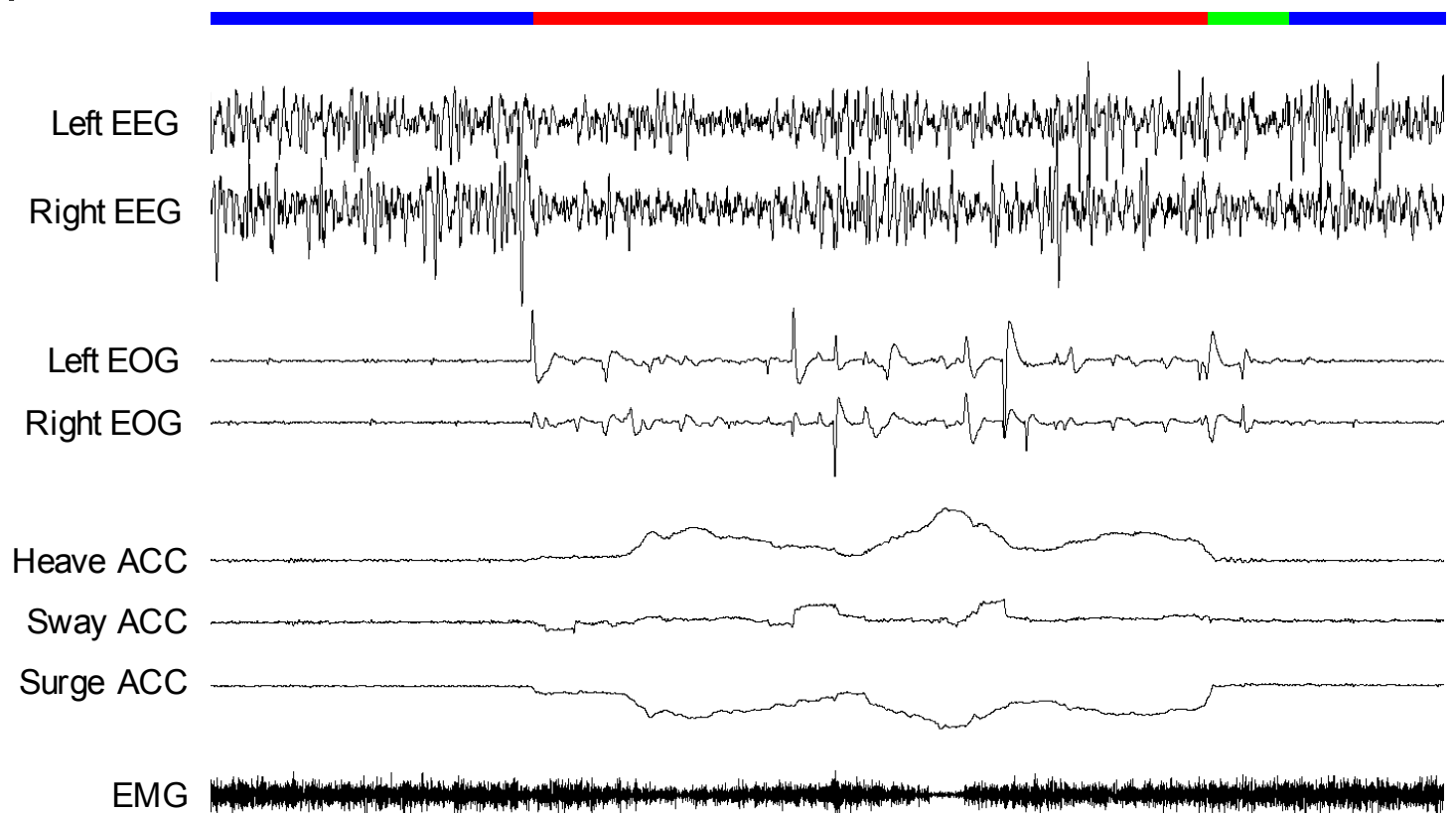

G

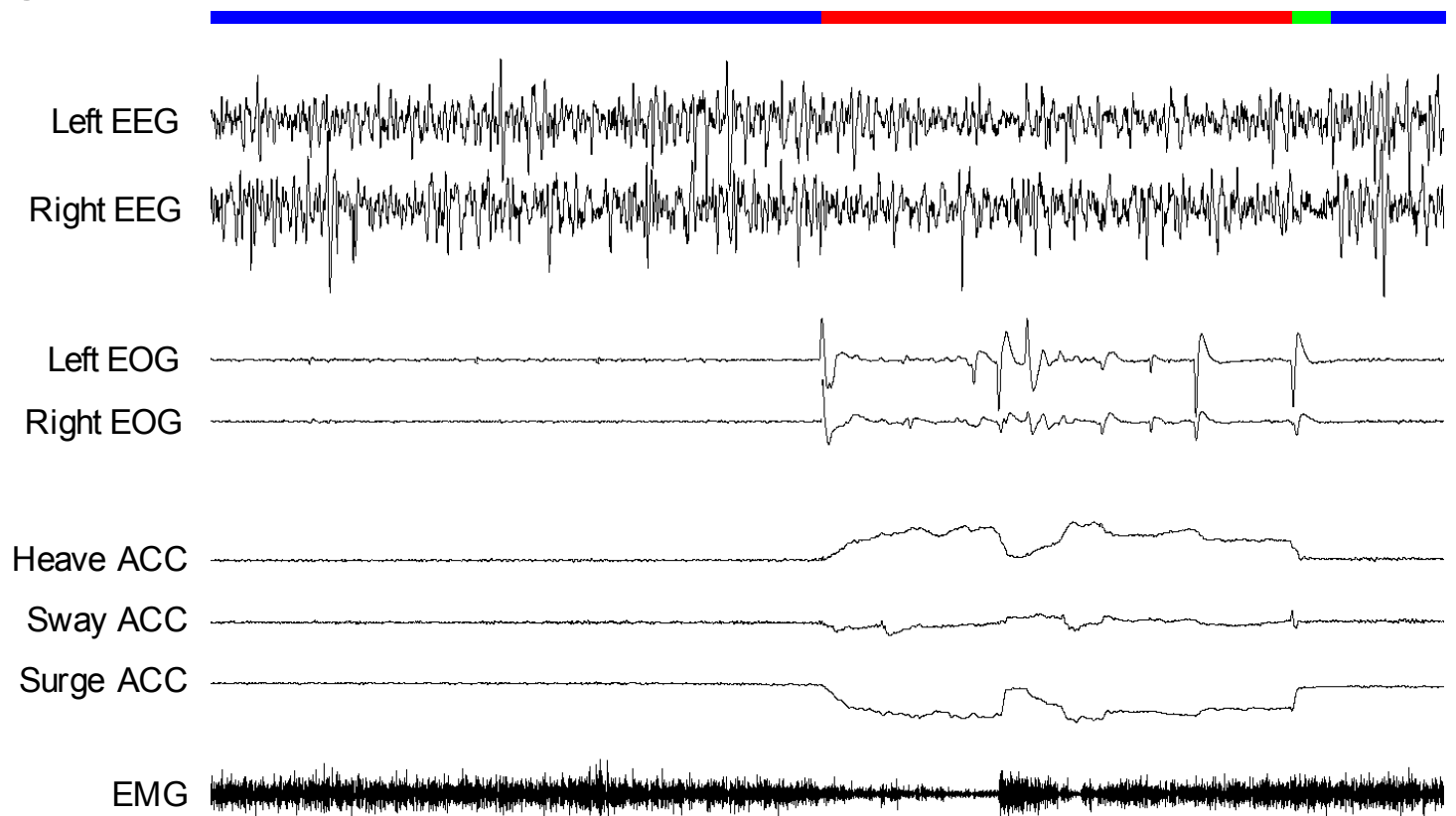

H

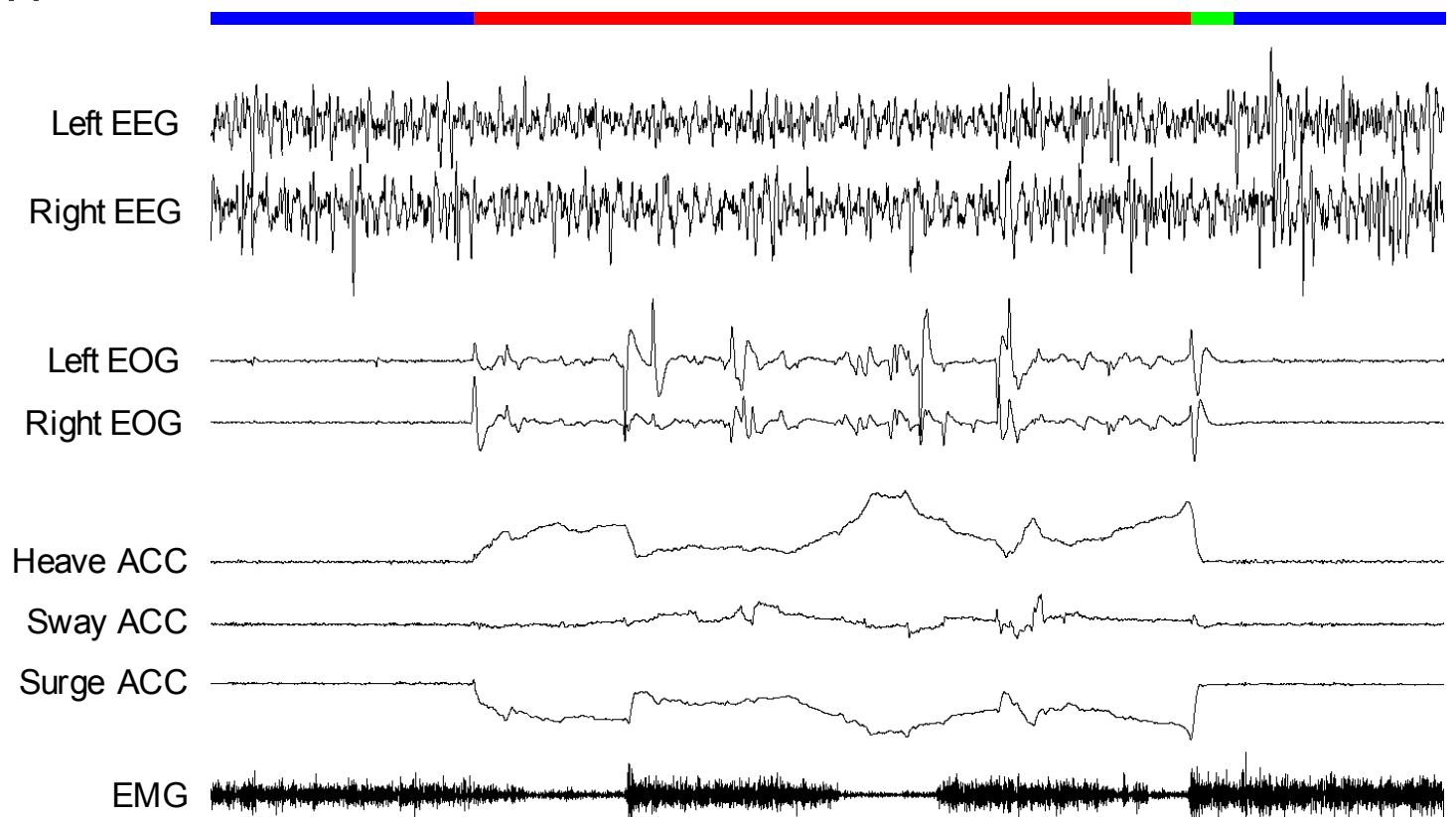

Supplement: Figure S1 — (A–H) Electroencephalogram (EEG) of the left and right hyperpallia, electrooculogram (EOG) of the left and right eye, the three axes (heave, sway and surge) of the head-mounted accelerometer (ACC), and electromyogram (EMG) of the nuchal muscle showing slow wave sleep (SWS, blue bar), rapid eye movement (REM) sleep (red bar) and wakefulness (green bar) in the ostrich. See main text for a description of each state. These figures illustrate the well-defined nature of an episode of REM sleep, as well as demonstrate the variation in EEG and EMG activity during REM sleep. Heave ACC: movement along the dorso-ventral axis with a positive slope denoting downward movement, Sway ACC: lateral axis with positive denoting movement to the right, Surge ACC: anterior-posterior axis with negative denoting movement forward. Vertical bars to the right of each EEG, EOG and EMG trace denote 100 µV, and 100 milli g-forces to the right of each ACC trace. Trace duration: 60 s. (PDF) [file pone.0023203.s001.pdf]
