# Supplementary material for: Ostriches Sleep like Platypuses
Source: PLoS One. 2011 Aug 24;6(8):e23203. doi: 10.1371/journal.pone.0023203 (PMC3160860; doi:10.1371/journal.pone.0023203)
Supplement: Text S1 — Supporting Material. (PDF) [file pone.0023203.s004.pdf]

Supporting Material for “Ostriches Sleep Like Platypuses”

Lesku JA, Meyer LCR, Fuller A, Maloney SK, Dell’Omo G, Vyssotski AL, Rattenborg NC (2011) Ostriches sleep like platypuses. *PLoS ONE*, doi:10.1371/journal.pone.0023203.

Surgical Details and Logger Specifications

Before surgery, each animal was given a local anesthetic (5 ml 2% lidocaine with adrenaline; Bayer, South Africa) subcutaneously in the surgical field, a non-steroidal anti-inflammatory (I.M. 10 mg / kg meloxicam, MOBIC®; Boehringer Ingelheim, South Africa) and a broad spectrum antibiotic (I.M. 10 mg / kg enrofloxacin, Baytril®; Bayer, South Africa). Throughout the procedure, heart and respiratory rate, oxygen saturation, and colonic temperature were monitored. Animals were anesthetized with isoflurane (induction and maintenance at 8% and 2 – 5%, respectively, vaporized in 100% oxygen) administered initially via facemask then an endotracheal tube. Four holes (0.5 mm diameter) were drilled through the exposed cranium to the level of the dura. Holes were arranged symmetrically over the left and right hyperpallia (comparable to the primary visual cortex of mammals) [1], a particularly prominent brain region in ostriches [2]. The holes were located 18 mm and 8 mm anterior to the parieto-occipital suture (lambda) and 6 mm lateral of the midline. The positioning of electrodes on the hyperpallium was facilitated through the examination of dead specimens of similar size. A fifth hole was drilled 13 mm anterior of lambda over the left hemisphere for the ground. Electroencephalogram (EEG) electrodes consisted of gold-plated round-tipped pins (0.5 mm diameter). Stainless steel wire electrodes were glued to the anterior and posterior margin of the supraorbital ridge over both eyes for the electrooculogram (EOG); two wires were sutured to the nuchal (neck) muscle for the electromyogram (EMG). All wires terminated at a plug housed in an aluminum box (length x width x height: 44 x 24 x 32 mm) secured over the center of the cranium with dental acrylic. The plug connected to an upgraded version of a logger (Neurologger) previously used for recording the EEG of birds [3] (www.vyssotski.ch/neurologger.html). Upgraded features include (i) the ability to record accelerations of the head, (ii) increased maximum recording duration and (iii) lower power consumption. A 3-dimensional accelerometer (MMA7260QT; Freescale Semiconductor Inc., U.S.A.) on the Neurologger recorded acceleration along each axis. To increase maximum recording duration, the previously used 1 GB Secure Digital (SD) memory card was replaced with a lighter, 8 GB microSD card. Voltage on the board was reduced from 3.3 V to

2.7 V, and the frequency of the processor was lowered from 24 MHz to 16 MHz, such that the modified logger consumed only 4.5 mA. Memory card and batteries were renewed every 8 – 10 d. The logger digitized the eight channels (2 EEG, 2 EOG, 1 EMG and 3 accelerometer) at 1600 Hz and stored averaged band-pass filtered (1 – 240 Hz) values at 800 Hz. For hypothalamic brain temperature measurements, one hole (2 mm diameter) was drilled 28 mm anterior of lambda to the level of the dura through which a ruggedized glass-coated bead thermistor (30 mm length, 2 mm outer diameter; Thermometrics, U.S.A.) was inserted, as per our previous study on brain temperature in ostriches [4]. Thermistors have been similarly implanted in other studies of avian sleep [5,6]. Our thermistor was connected to a logger positioned subcutaneously in the neck. At the end of the study, all equipment was removed from the birds using similar surgical procedures to those outlined above, and the animals were returned to the reserve following post-operative recovery in the outdoor enclosures.

References

1. Medina L, Reiner A (2000) Do birds possess homologues of mammalian primary visual, somatosensory and motor cortices? Trends Neurosci 23: 1-12.
2. Corfield JR, Wild JM, Hauber ME, Parsons S, Kubke MF (2008) Evolution of brain size in the Palaeognath lineage, with an emphasis on New Zealand ratites. Brain Behav Evol 71: 87-99.
3. Vyssotski AL, Serkov AN, Itskov PM, Dell'Omo G, Latanov AV, et al (2006) Miniature neurologgers for flying pigeons: multichannel EEG and action and field potentials in combination with GPS recording. J Neurophysiol 95: 1263-1273.
4. Fuller A, Kamerman PR, Maloney SK, Mitchell G, Mitchell D (2003) Variability in brain and arterial blood temperatures in free-ranging ostriches in their natural habitat. J Exp Biol 206: 1171-1181.
5. Gusel'nikova EA, Pastukhov YF (2009) Microinjection of 70-kDal heat shock protein into the oral reticular nucleus of the pons suppresses rapid eye movement sleep in pigeons. Neurosci Behav Physiol 39: 289-296.
6. Szymczak JT, Narebski J, Kadziela W (1989) The coupling of sleep-wakefulness cycles with brain temperature of the rook, *Corvus frugilegus*. J Interdiscip Cycle Res 20: 281-288.
